# Reactive balance to unanticipated trip-like perturbations: a treadmill-based study examining effect of aging and stroke on fall risk

**DOI:** 10.1080/23335432.2018.1512375

**Published:** 2018-09-21

**Authors:** Mansi Joshi, Prakruti Patel, Tanvi Bhatt

**Affiliations:** Department of Physical Therapy, University of Illinois at Chicago, Chicago, IL, USA

**Keywords:** Chronic stroke, dynamic stability, reactive balance, postural control

## Abstract

The purpose of this study was to examine the mechanism of fall risk in community-dwelling ambulatory hemiplegic stroke survivors when exposed to a sudden, trip-like support surface perturbation in standing. Participants (*n* = 14 / group) included stroke survivors, Age-similar older controls (AC), and Young controls (YC) experienced trip-like perturbation on a motorized treadmill. The primary outcomes were COM state control (measured as COM position (X_COM/BOS_) and velocity (V_COM/BOS_) relative to the base of support (BOS)) and the vertical limb support recorded as the extent of hip descent. All participants demonstrated forward loss of balance (FLOB) followed by an equal first compensatory step length. At step touchdown, stroke survivors demonstrated lower COM state stability and increased trunk flexion than the YC group. Stroke survivors also demonstrated greater hip descent than YC and AC groups, as they first stepped with their non-paretic limb. For the second compensatory step, the stroke survivors stepped with their paretic limb. However, unlike the AC group, they were unable to control V_COM/BOS_ despite a longer compensatory step. In conclusion, poor control of COM state, impaired trunk control and inability of the paretic limb to provide vertical limb support may explain the higher fall-risk in stroke survivors.

## Introduction

About 3.4 million individuals above 18 years of age are predicted to encounter an episode of stroke by the year 2030 which is a 20.5% increase in prevalence from the year 2012 (Mozaffarian et al. 2015). Stroke is associated with a higher fall risk even through the chronic phase (Mackintosh et al. 2005). It is also a key contributor to long-term disability and impairment (O’Sullivan et al. 2013). With regards to increasing concern about fall risk in this population, it is essential to understand the underlying mechanisms of falls to develop effective intervention programs for reducing falls risks.

Community-dwelling ambulatory hemiplegic stroke survivors in the chronic phase continue to exhibit residual sensorimotor impairments such as delayed postural muscle responses (Marigold and Eng 2006), weight-bearing asymmetry and impaired inter-limb coordination (Marigold et al. 2004). These may contribute to poor balance control during dynamic balance tasks (Geurts et al. 2005) and community ambulation (Keenan et al. 1984). Thus, community-dwelling ambulatory hemiplegic stroke survivors are at risk of balance loss while navigating in the community.

Fall incidence in the community can occur due to slips and trips based on perturbation direction (Robinovitch et al. 2013). These perturbations challenge one’s postural stability and induce balance loss by displacing the center of mass (COM) state beyond the limits of the base of support (BOS) (Maki and McIlroy 1997). One can usually recover from such balance loss and regain stability of their COM state through an instantaneous compensatory stepping response. Furthermore, the compensatory stepping response combined with vertical limb support through adequate joint moment during single-support and double-support phases of compensatory stepping would prevent a collapse following perturbation (Pai et al., 2006; Yang et al. 2009).

Recent studies examining COM state stability (i.e. COM  position and velocity relative to BOS) during compensatory stepping in response to a slip-induced perturbation in standing have shown a lower COM state stability in chronic stroke survivors as compared with age-similar controls at compensatory step touchdown (Patel and Bhatt 2016; Salot et al. 2016); thus, suggesting greater instability in the backward direction in even after executing a recovery response. While slip-related responses are important, chronic stroke survivors are equally predisposed to trip-like environmental perturbations (Schmid et al. 2013), which displaces the COM state forward relative to BOS. Recovery from such responses requires efficient and effective forward stepping to extend the BOS to control the forward trunk momentum (Pavol et al. 2001; Crenshaw et al. 2012).

Laboratory studies focusing on trip-like perturbations in older adults have reported use of a multiple stepping strategy (Carty et al. 2011), delayed and short first recovery step (Pavol et al. 2001), and increased trunk flexion angle and velocity (Pavol et al. 2001; Carty et al. 2011) as factors contributing to increased fall risk in this population. Additionally, previous comparative studies between older and young individuals have identified decreased limb support (Pijnappels et al. 2004), an anterior COM at the instant of trip recovery at touchdown (Van Dieen et al. 2005) as factors related to trip-induced falls during overground and treadmill-based walking. There is considerable literature examining compensatory stepping responses to trip-like perturbations in healthy older and young individuals (Carty et al. 2011; Wang et al. 2012). However, there is a lack of literature examining trip-related responses in chronic stroke survivors.

The study’s purpose, therefore, was to examine the underlying biomechanical factors predisposing chronic stroke survivors to falls compared to their healthy counterparts, when subjected to sudden, trip-like support surface perturbation while standing. We hypothesized that upon the unexpected perturbation, the influence of stroke would be associated with a reduced COM state stability resulting from decreased vertical limb support, affecting efficient compensatory stepping when compared with healthy age-similar adults. Since majority of the recruited stroke survivor sample included older adults (>55 years old), to further verify that the changes in postural and stepping parameters demonstrated were indeed due to the influence of stroke not aging, both groups were compared with healthy young controls (YC). If both the stroke survivors and healthy controls differed from the young control, with no difference between the stroke survivors and age-control groups, the observed changes could be attributed to an aging effect rather than stroke.

## Materials and methods

This cross-sectional study included 14 YC, age-similar older controls (AC), and community-dwelling ambulatory hemiplegic stroke survivors (demographic details: Table 1). Stroke survivors were included if their physicians confirmed diagnosis of stroke (>6 months poststroke (Vivian Weerdesteyn et al. 2008)) and demonstrated the ability to stand independently without any assistive device. Inclusion criteria for control groups were an absence of any self-reported medical condition. The study was conducted at the University of Illinois at Chicago (UIC). Preceding participation recruited participants signed an informed consent by UIC, Institutional Review Board (IRB).

**Table 1.** Demographic and clinical characteristics of stroke survivors and age-similar older controls (AC) groups. CMSA = Chedoke McMaster Stroke Assessment, MOCA = Montreal Cognitive Assessment, TUG = Timed up and go test, BBS = Berg balance scale, n/a = not applicable

Participant characteristics	Stroke (*n* = 14)	Age-similar older controls (*n* = 14)	Young controls (*n* = 14)
Age(y), x̄ (SD)	60.07 (8.06)	58.57 (6.14)	24.28 (3.70)
Sex (Male/Female), N	7/7	4/10	5/9
Weight (kg), x̄ (SD)	172.42 (23.40)	166.28 (35.88)	153.28 (25.47)
Height (cm), x̄ (SD)	169.71 (7.60)	168.23 (11.06)	170.34 (7.92)
Foot length (cm), x̄ (SD)	26.18 (1.65)	25.77 (1.53)	25.84 (1.73)
Hemiparetic side (left/right), N	6/8	n/a	n/a
Type of stroke, N			
A. Ischemic	6/14	n/a	n/a
B. Hemorrhagic	7/14	n/a	n/a
C. Transient Ischemic Attack	1/14	n/a	n/a
Location of stroke, N			
(A) Cortical Stroke	14/14		
Time since stroke (y), x̄ (SD)	9.07 (5.49)	n/a	n/a
BBS (/56), x̄ (SD)	43.6 (6.54)	51.2 (3.51)	n/a
TUG (s), x̄ (SD)	15 (4.57)	n/a	n/a
MOCA (/30), x̄ (SD)	25.5 (2.53)	n/a	n/a
CMSA (/7) Median (Range)	5 (3–6)	n/a	n/a
No. of stroke survivors using orthoses	10/14	n/a	n/a
No. of stroke survivors using assistive device	12/14	n/a	n/a

### Reactive balance testing protocol

Participants were subjected to a sudden, trip-like backward directed perturbation in standing position on a motorized treadmill (ActiveStep, Simbex, Lebanon, New Hampshire). The participants were initially subjected to a single familiarization trial with the purpose of exposing the participant to a novel trip-like perturbation at 0.67m/s for 0.19 m with an acceleration of 16.75m/s^2^. This was followed by each participant receiving only a single perturbation testing trial (that was used for analysis) at a higher intensity of 0.77 m/s for 0.26 m with an acceleration of 19.24 m/s ^2^. Only one trial was given to examine the true reactive response and eliminate any adaptive responses over repeated trials. The participant performed both the trials in standing position. The data was analyzed from the testing trial. The onset time for each perturbation was randomized and occurred 5–20 s after the participant assumed their starting position.

**Figure 1.**
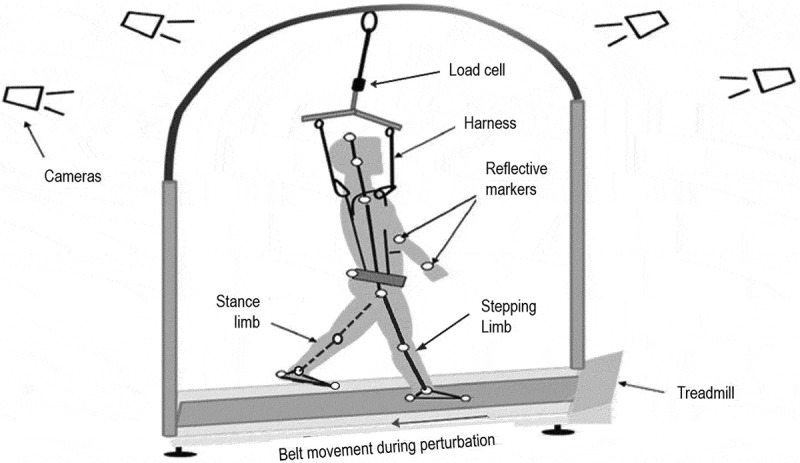
Schematic representation of the experimental set up. The white circles represent the marker positions and the arrow shows the direction of belt movement during perturbation. The protocol design consisted of familiarization trial, which initially exposed the participants to a novel trip-like perturbation followed by single perturbation testing trial (considered for analysis). Participants were instructed to demonstrate a natural response after assuming a comfortable stance on the treadmill, where they experienced a trip-like perturbation wherein the treadmill belt suddenly accelerated in the backward direction. In an event of a fall, a overhead safety harness prevented the participant’s knees from touching the treadmill belt. A load cell fixed in series with the safety harness, recorded the amount of force exerted on the harness in the downward direction post-perturbation onset

Safety harness suspended from the overhead treadmill arch prevented participant’s knees from touching the treadmill belt during a fall incidence. A load cell fixed in series with the safety harness recorded the amount of force exerted on the harness in the downward direction post-perturbation onset. The stroke survivors did not receive any instruction to correct any inherent weight-bearing asymmetry before the perturbation onset with the aim of examining their innate response to a sudden, trip-like perturbation (Figure 1).

### Data collection and analysis

An eight-camera motion capture system (Motion Analysis Corp, Santa Rosa, California) recorded full body kinematics with a sampling rate of 120 Hz. The Helen Hayes marker set comprising of 29 reflective markers was positioned on the bony landmarks, head, and trunk for computing the COM. One marker was placed on the treadmill belt to identify the perturbation onset. The marker data were processed through a low pass filter using a fourth-order butterworth filter with a cutoff frequency of 6 Hz. The load cell data were sampled at 1200 Hz. For stroke survivors, clinical assessment tools, such as Chedoke-McMaster Stroke Assessment (CMSA) leg impairment inventory, measured lower limb impairment and Montreal Cognitive Assessment (MOCA). Clinical assessment tools such as the Berg Balance Scale (BBS) and Timed Up & Go Test (TUG) were used to assess balance and level of impairment (Table 1).

### Outcomes

#### Perturbation outcome

The forward loss of balance (FLOB) was classified into a) a fall: occurred when the amount of body weight exerted on the safety harness exceeded 30% of the individual’s body weight (Yang and Pai 2011) and verified through visual inspection. b) recovery with a forward compensatory step. A forward compensatory step occurred when the stepping limb’s heel and toe demonstrated a clear liftoff (LO) and landed anterior to the non-stepping limb (Salot et al. 2016). The instance of LO and touchdown (TD) deduced by the Z coordinate of the toe or heel marker depending on the last point of contact for LO and first point of contact at TD.

#### Postural control

**Figure 2.**
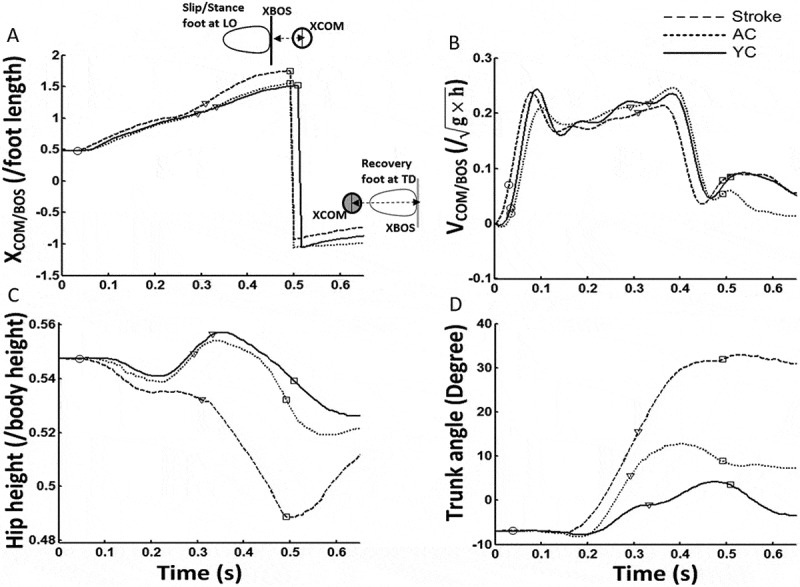
Figure showing traces of (a) XCOM/BOS, center of mass (COM) position relative to base of support (BOS), (b) VCOM/BOS, COM velocity relative to BOS, (c) Hip height, and (d) Trunk angle from a representative participant from the stroke, age-similar older control (AC) and young control (YC) groups. The time zero indicates onset of the perturbation

Posture control was examined by computing two key variables - the COM position (X_COM/BOS_) relative to the BOS and COM velocity relative to the BOS. Joint position data were used to compute the absolute COM position. The absolute COM  position was then expressed relative to the anterior margin of BOS (X_COM/BOS_) by obtaining the difference between the absolute COM position and BOS position. The stance limb toe at LO and the stepping limb toe at TD formed the anterior margin of BOS. The X_COM/BOS_ was normalized by the participant’s foot length (Patel and Bhatt 2016) (Figure 2(a)). The absolute COM velocity was calculated as the first-order derivative of the X_COM/BOS_ and then expressed relative to the velocity of the BOS by obtaining difference between the COM velocity and BOS velocity (V_COM/BOS_). The V_COM/BOS_ was then normalized by the dimensionless fraction of √*gh*, (*g* is the acceleration due to gravity and *h* is the individual’s body height) (McMahon 1984). The instantaneous X_COM/BOS_ and V_COM/BOS_ were subsequently extracted at specific time instances of LO and TD of the first and second compensatory steps (Figure 2(b)).

An anterior shift in X_COM/BOS_ and increase in V_COM/BOS_ predisposes one to a FLOB. V_COM/BOS_ at LO and TD, positive values of X_COM/BOS_ indicate that absolute COM was significantly more forward than the anterior margin of the BOS. While at LO under perturbed conditions the X_COM/BOS_ is expected to be positive, at TD the values could be positive or negative. At TD of the compensatory step, if the COM was located within the BOS (ideal recovery), the values would be negative. More negative values X_COM/BOS_ indicated that the COM was located well inside the BOS (i.e. posterior to the anterior margin of the BOS). Positive values at TD indicated that the X_COM/BOS_ was still forward to the BOS and would increase likelihood of a second compensatory step. Similarly, positive values of V_COM/BOS_ at LO and TD indicate that the COM is traveling faster than the anterior margin of the BOS. Larger positive values indicate a greater difference between the forward velocities of the COM and BOS. Negative values indicate that V_COM/BOS_ indicate that the COM is traveling behind the anterior margin of the BOS. A value of zero indicates that both the COM and BOS are traveling at the same velocity.

### Vertical limb support

Vertical limb support was recorded as the extent of hip descent from standing (i.e. maximum vertical descent (peak Z hip) of the midpoint of the hip) after the perturbation onset until TD of the first compensatory step (Figure 2(c)). The midpoint of the hip was obtained from the right and left ASIS markers. The Z hip was normalized to the individual’s body height (Yang et al. 2009).

#### Compensatory step and trunk kinematics

The time difference between the perturbation onset until LO of the stepping limb was identified as step initiation time (seconds) (Salot et al. 2016). The step execution time (seconds) was defined as the time difference between the LO and TD of the stepping limb (Salot et al. 2016). Compensatory step length was recorded to the extent of stepping limb heel displacement from LO to TD for the first and the second compensatory step. Further, the compensatory step length was normalized by the individual’s body height (Salot et al. 2016). The numbers of compensatory steps executed to recover balance were also recorded.

Trunk angle was measured as the trunk orientation from the vertical position in the sagittal plane (Patel and Bhatt 2016). The reflective markers at C7 (spinous process of seventh cervical vertebra) and S2 (spinous process of second sacral vertebra) and the two shoulder markers represented the sagittal plane of the trunk. Thus, zero degree represents the vertical orientation of the trunk, a more positive value for trunk angle indicated greater trunk flexion and vice versa. The instantaneous trunk angle was obtained at the time events of LO and TD of the first compensatory step (TD step 1) (Figure 2(d)). The peak trunk angle (i.e. maximum trunk flexion angle) was recorded post-perturbation onset between LO and TD. To normalize for any deviations from vertical, trunk angle at these instances was expressed relative to the trunk angle in normal stance before perturbation onset.

## Statistical analysis

The Kruskal-Wallis test analyzed the effect of external perturbation on proportion of falls and number of steps, thereafter followed up with Mann-Whitney U test. The Shapiro–Wilk test was performed to examine the normality of the parametric outcome variables. After normality was tested and not violated, a 3 × 2 two-way analysis of variance (ANOVA) was performed for the first compensatory step to compare the differences in X_COM/BOS,_ V_COM/BOS_ and trunk angle from LO to TD between the three groups. Significant main effects [main effect of event (LO & TD) and main effect of group (YC, AC, and stroke survivors)] and interactions were resolved using *post hoc t*-tests (paired for within group and unpaired for between group). Bonferroni correction was applied to adjust the p-value to account for multiple comparisons. A one-way ANOVA was performed to analyze the differences between the groups for first compensatory step length and peak trunk angle. Significant main effects were resolved using Bonferroni *post hoc* test. The normality was violated for the number of steps (W = 0.63, *p* = 0.00), step initiation time (W = 0.005, *p* = 0.005) and vertical limb support (W = 0.90, *p* = 0.03) and accordingly Kruskal-Wallis test was used to analyzed differences in these variables between the three groups and followed up with Mann Whitney U test.

To determine the relationship between trunk control and COM state stability (X_COM/BOS_ and V_COM/BOS_) at TD, a stepwise backward multiple regression was performed for each group with X_COM/BOS_ at TD as the dependent variable and trunk angle at TD and compensatory step length as independent variables. Similarly, a linear regression was performed with V_COM/BOS_ at TD as dependent variable and with trunk velocity at TD as independent variable.

A two-way ANOVA was performed to compare and analyze the differences in X_COM/BOS_ and V_COM/BOS_ from TD of first compensatory step to TD of second compensatory step between the stroke survivors and AC group. Significant main effects and interactions were resolved using paired and unpaired *post hoc t*-tests. An independent t test was performed to determine the between group difference for the second compensatory step length. Thirteen subjects from the AC group and 10 subjects from the stroke survivors group initiated a second compensatory step and were considered for second compensatory step analyses. Only 8 out of 14 YC initiated a second compensatory step. The YC group was not considered for the second step analyses, although their data is present in the results section.

To determine the relationship between motor disability and COM state stability (X_COM/BOS_ and V_COM/BOS_), a stepwise backward multiple regression analyses was performed between TUG and BBS as dependent variables and COM state stability (X_COM/BOS_ and V_COM/BOS_) as independent variables. A Spearman’s rank correlation was performed to analyze the relationship between CMSA (dependent variable) and COM state stability (X_COM/BOS_ and V_COM/BOS_) (independent variables).

## Results

### Perturbation outcome

**Figure 3.**
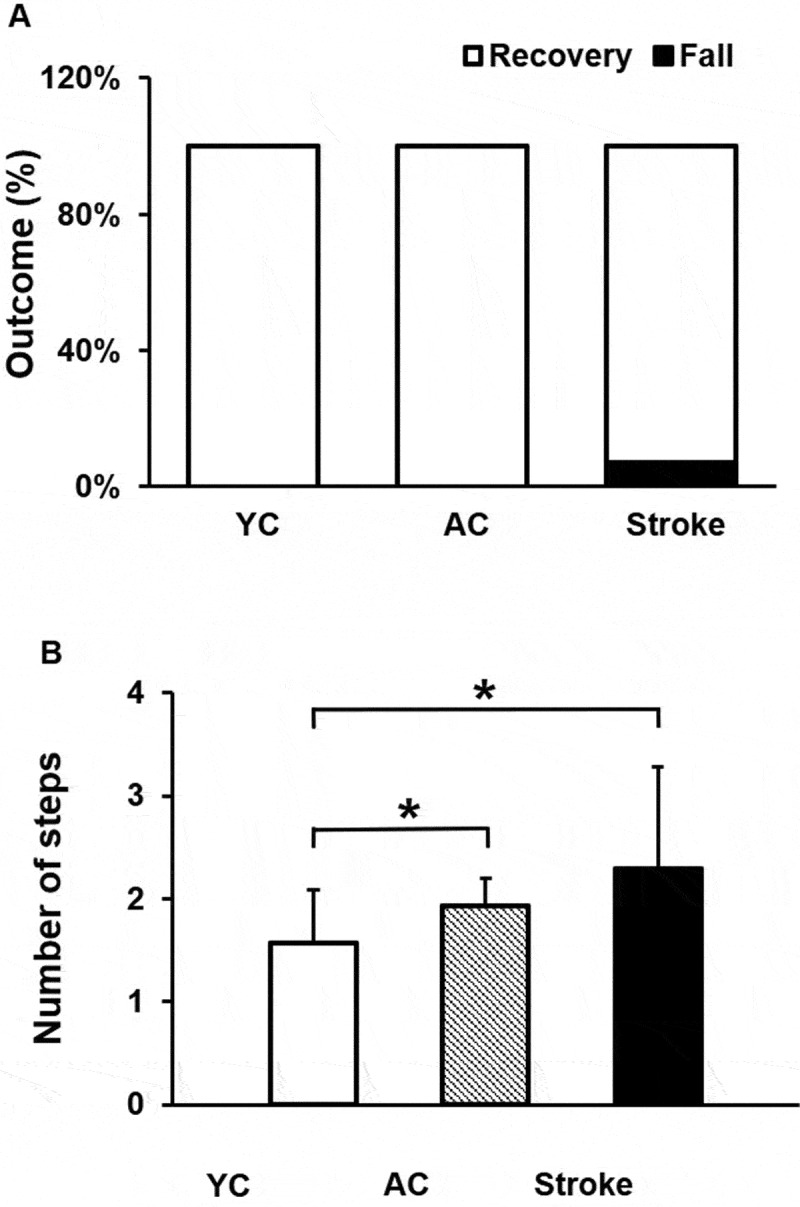
Results representing the perturbation outcome in response to trip-like perturbation between young controls (YC), age-similar older controls (AC) and stroke survivors for (a) Percentage outcome of falls vs. recovery. About 92.8% of stroke survivors executed the first compensatory step with their paretic limb and 7.2% executed their first step with paretic limb. About, 42.85% – YC and 50% – AC groups executed their first compensatory step with their dominant limb. (b) Number of compensatory steps were executed.”

All participants experienced FLOB and initiated a forward compensatory stepping response resulting in a fall or a recovery (Figure 3(a)). There was no significant difference among the groups with regards to the incidence of falls, χ^2^ = 2.00, *p* = 0.36. Majority of stroke survivors (13/14) initiated the first compensatory step with their non-paretic limb. A significant difference among the groups was observed for the number of compensatory steps, χ^2^ = 2.00, *p* = 0.04. Stroke survivors (U = 56, *p* = 0.04), and AC group (U = 63, *p* = 0.03), demonstrated significantly more number of compensatory steps as compared with the YC group (Figure 3(b)).

### First compensatory step

**Figure 4.**
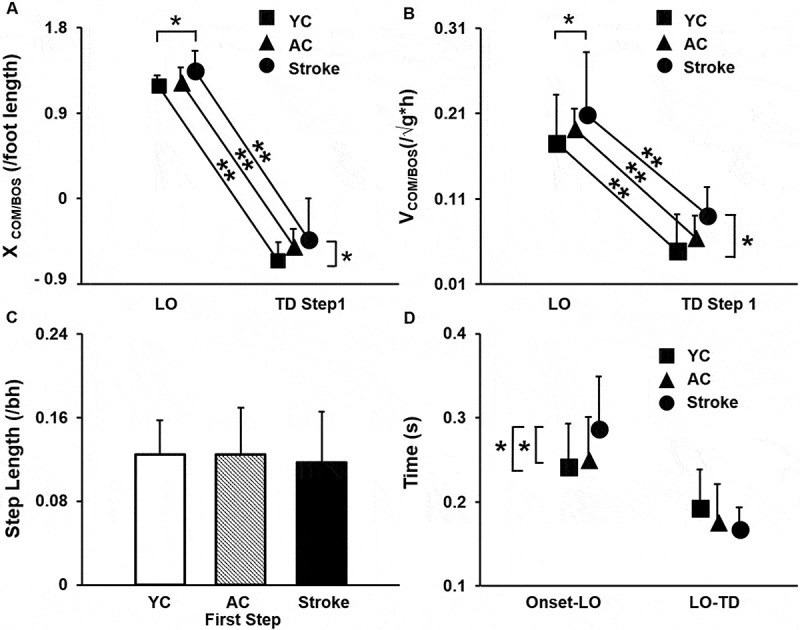
Mean (SD) differences among young controls (YC), age-similar older controls (AC) and stroke survivors for (a) Center of mass position relative to base of support (XCOM/BOS) at first step liftoff (LO) and touchdown (TD), (b) Center of mass position velocity relative to heel velocity (VCOM/BOS) at first step LO and TD, (c) First compensatory step and (d) Step initiation time (time elapsed between the belt onset and LO) and step execution time (time elapsed between LO to TD). The XCOM/BOS was normalized to the individuals’ foot length and VCOM/BOS was normalized to the square root of gravity * body height (√gh).Positive values for XCOM/BOS and VCOM/BOS indicates that the COM is anterior to and traveling faster in the forward direction relative to the BOS. Negative values for XCOM/BOS indicate that the COM is posterior to the anterior margin of BOS. Negative values for VCOM/BOS indicate that the COM is traveling slower than the BOS. Significant differences within and between the groups are indicated by * *p* < 0.05 and ** *p* < 0.01. Note: The data presented includes 14 participants in each group

For the first compensatory step, there was a significant main effect of event (first step LO and TD) for X_COM/BOS_ [F_1, 39_ = 1172.29, *p* < 0.001] (Figure 4(a)) and V_COM/BOS_ [F_1, 39_ = 222.72, *p* < 0.001] (Figure 4(b)). However, no significant event (first step LO and TD) x group (YC, AC, and Stroke) interaction was observed for X_COM/BOS_ [F_2, 39 =_ 0.49, *p* = 0.61] and V_COM /BOS_ [F_2, 39_ = 0.11, *p* = 0.88]. A significant main effect of group was present for X_COM/BOS_ [F_2, 39_ = 4.49, *p* = 0.01] and V_COM /BOS_ [F_2, 39_ = 3.64, *p* = 0.03]. Furthermore, *post hoc* analysis revealed a significantly more anterior X_COM/BOS_ and faster moving V_COM/BOS_ at LO and TD in the stroke survivors compared with YC group [X_COM/BOS_ (*p* = 0.01, 95% CI = 0.03, 0.33); V_COM/BOS_ (*p* = 0.03, 95% CI = 0.002, 0.07)]. Although, no significant difference was observed at LO and TD for both X_COM/BOS_ and V_COM/BOS_ between stroke survivors and AC groups [X_COM/BOS_ (*p* = 0.41, 95% CI = −0.06, 0.24); V_COM /BOS:_ (*p* = 0.42, 95% CI = −0.01, 0.05)] and YC & AC groups [X_COM/BOS_ (*p* = 0.43, 95% CI = 0.03, 0.33); V_COM /BOS_ (*p* = 0.43, 95% CI = −0.24, 0.06)].

There was no difference in the first compensatory step length between the three groups [F_2, 41_ = 0.15, *p* = 0.85] (Figure 4(c)). The step initiation time, however, demonstrated significant group difference [χ^2^ = 2.00, *p* = 0.02]. *Post hoc* tests revealed that the stroke survivors exhibited a significant delay in the first compensatory step initiation than the YC (U = 42, *p* = 0.01) and AC (U = 51, *p* = 0.03). The step execution time did not differ among the groups [F_2, 41_ = 1.22, *p* = 0.30] (Figure 4(d)).

**Figure 5.**
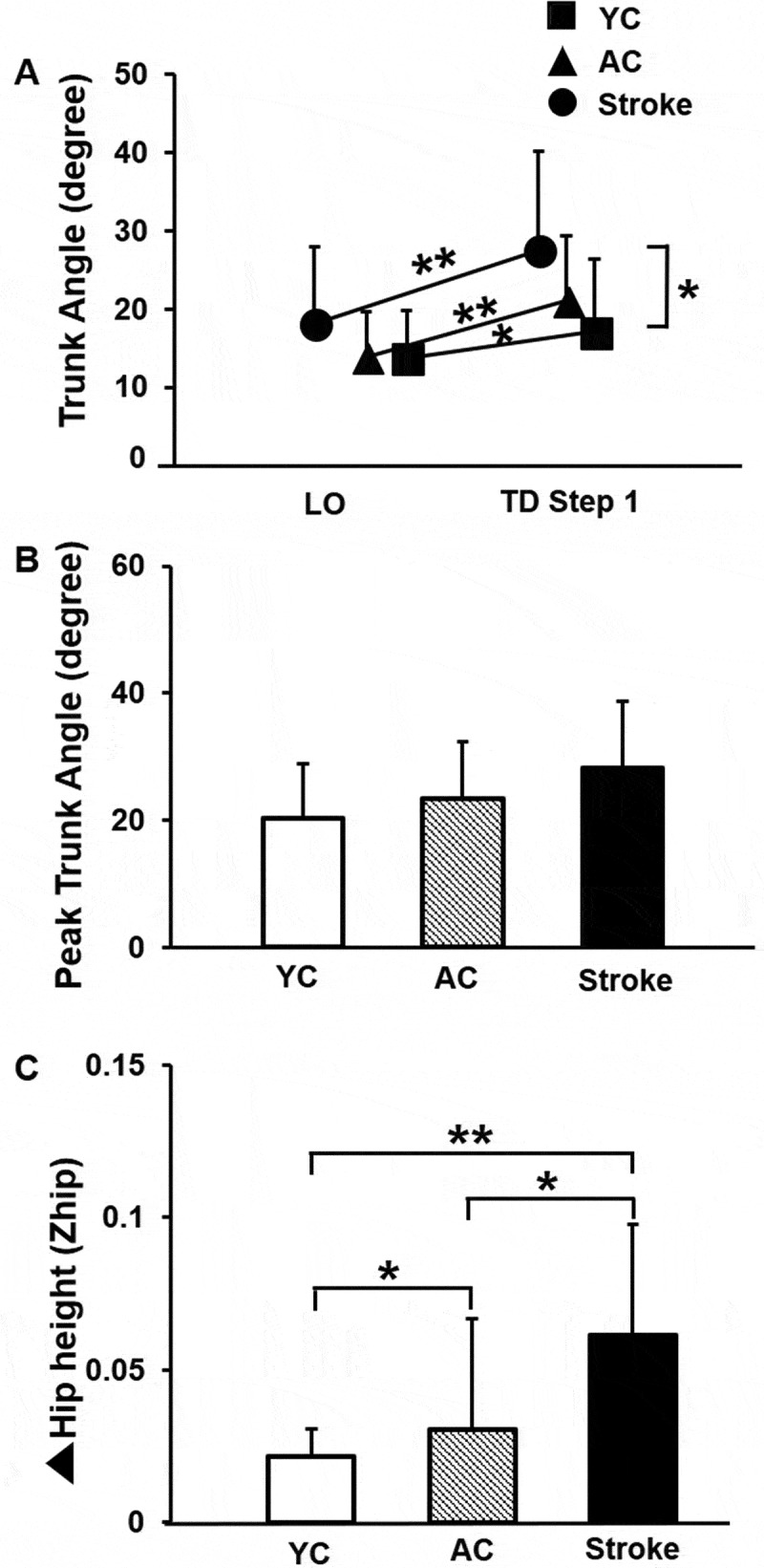
Mean (SD) differences among young controls (YC), age-similar older controls (AC) and stroke survivors for (a) Trunk angle from the first step liftoff (LO) to touchdown (TD) and (b) Peak trunk angle. Larger values indicate greater trunk flexion. (c) Δ Hip height (Z hip) measured change in hip height at TD relative to pre-perturbation, stance hip position. Larger Z hip values indicate greater hip descent. Significant differences within and between groups are indicated by * *p* < 0.05 and ** *p* < 0.01

There was a significant difference in trunk angle across the groups (YC, AC, and stroke survivors) from LO to TD of the first compensatory step (significant event x group interaction [F_2, 39_ = 3.79, *p* = 0.03]) (Figure 5(a)). A significant main effect of event from LO to TD for the first compensatory step [F_1, 39_ = 53.44, *p* < 0.001] was observed. However, main effect of group was not significant [F_2, 39_ = 2.91, *p* = 0.06]. Each group increased trunk flexion from LO to TD [YC: *p* = 0.04, 95% CI = −6.51, −0.12; AC: *p* < 0.001, 95% CI = – 9.61, −4.87; Stroke survivors: *p* < 0.001, 95% CI = −13.69, −5.01]. At LO, there was no between group difference in trunk angle (YC vs. AC: *p* = 0.96, 95% CI = −4.66, 4.44; AC vs. stroke survivors: *p* = 0.16, 95% CI = −10.37, 1.87; YC vs. stroke survivors: *p* = 0.15, 95% CI = −10.54, 1.82). At TD, the trunk angle was greater (increased flexion) for the stroke survivors compared with YC group (*p* = 0.02, 95% CI = −19.01, −1.77). However, no difference in trunk angle (flexion) at TD was observed between YC and AC and stroke survivors [YC vs. AC: *p* = 0.23, 95% CI = −10.85, 2.77; AC vs. stroke survivors: *p* = 0.12, 95% CI = −14.65, 1.94]. Furthermore, the peak trunk angle also did not differ among the groups [F _2, 41_ = 2.51, *p* = 0.09] (Figure 5(b)). A significant difference in peak Z hip between the three groups was observed (χ^2^ = 17.02, *p* < 0.001) (Figure 5(c)), with the stroke survivors exhibiting the greatest hip descent from baseline compared with the control groups (stroke > AC > YC) (stroke survivors vs. YC: U = 21, *p* = 0.00; stroke survivors vs. AC: U = 51, *p* = 0.03; AC vs. YC: U = 51, *p* = 0.03).

**Figure 6.**
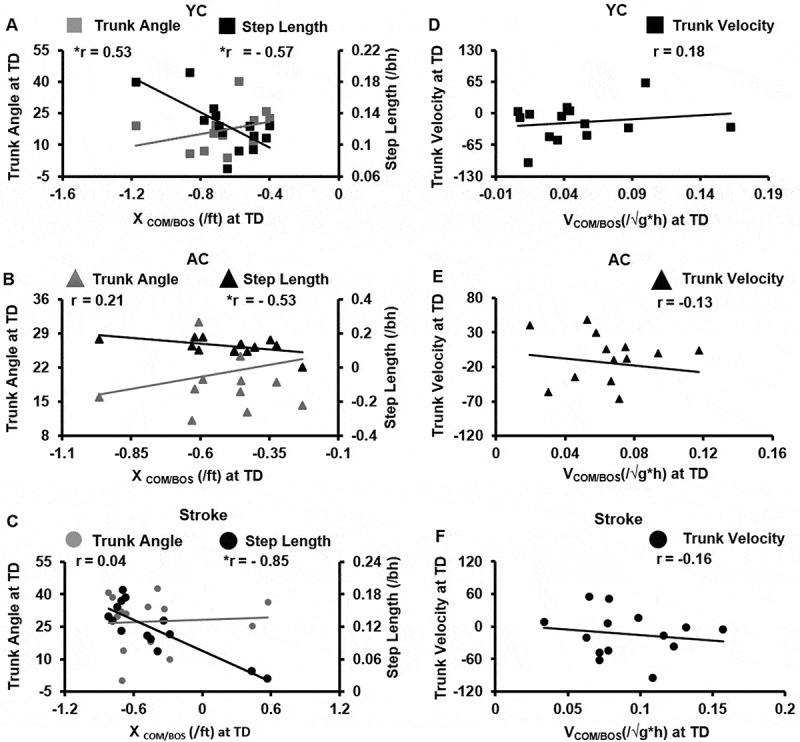
Scatter plots for young controls (YC), age-similar older controls (AC) and stroke survivors illustrating (a-c): Relationship between XCOM/BOS at TD and Trunk angle at TD and compensatory step length for young controls (YC), age-similar older controls (AC) and stroke survivors, respectively; (d-f): Relationship between VCOM/BOS at TD and Trunk Velocity at TD for young controls (YC), age-similar older controls (AC) and stroke survivors, respectively. Significance is indicated by * *p* < 0.05 and ** *p* < 0.01

Regression analysis examining relationship of COM state and trunk control in each group revealed differential results. Within the young adults group, X_COM/BOS_ at TD showed a significant negative correlation with compensatory step length (*r *= −0.57, *p *= 0.02) and significant positive correlation with the trunk angle at TD (*r *= 0.53, *p *= 0.03). The multiple regression model showed that only compensatory step length predicted X_COM/BOS_ at TD (*R^2^ *= 0.32, *p* = 0.04) (Figure 6(a)). Among the age-similar controls and stroke survivors, the X_COM/BOS_ at TD showed a significant negative correlation with compensatory step length (*r* = −0.53, *p* = 0.02 for age-similar control and *r* = −0.85, *p* < 0.001 for stroke survivors) but not with trunk angle at touchdown (*r* = 0.21, *p* = 0.22 for age-similar control and *r* = 0.06, *p* = 0.82 for stroke survivors) (Figure 6(b,c)). Further, for the AC and stroke survivors the X_COM/BOS_ at TD was significantly predicted by compensatory step length (*R^2^* = 0.29, *p* = 0.04 for age-similar control and *R^2^* = 0.72, *p* < 0.001 for stroke survivors). There was no significant correlation between trunk velocity at TD and V_COM/BOS_ at TD individually for all the three groups [YC (R^2^ = 0.03, *p* = 0.53; *r *= 0.18, *p* = 0.53); AC (R^2^ = 0.01, *p* = 0.64; *r *= −0.13, *p* = 0.64) and stroke survivors (R^2^ = 0.02, *p* = 0.58; *r *= −0.16, *p* = 0.58)] (Figures 6(d-f)).

### Second compensatory step

**Figure 7.**
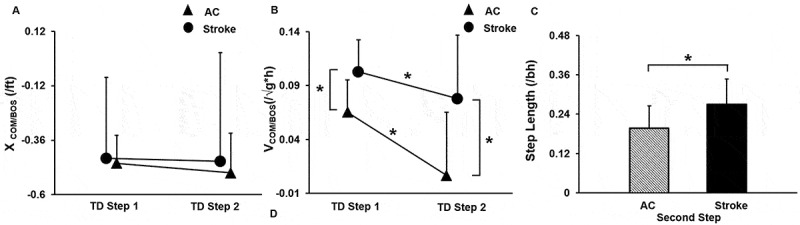
Mean (SD) differences between first and second step touchdown (TD) among the age-similar older controls (AC) and stroke survivors for (a) Center of mass position relative to base of support (XCOM/BOS), (b) Center of mass velocity relative to base of support heel velocity (VCOM/BOS), (c) Compensatory step length for second. The XCOM/BOS was normalized to the individuals’ foot length and VCOM/BOS was normalized to the square root of gravity * body height (√gh). Significant differences within and between groups are indicated by * *p* < 0.05 and ** *p* < 0.01. Note: The data presented includes 13/14 participants – AC group and 10/14 participants – stroke survivors who took a second compensatory step.”

The means for the COM state for YC participants who elicited a second compensatory step were: X_COM/BOS = −0_.35 ± 0.29, V_COM/BOS_ = −0.01 ± 0.03, respectively; with step length being −0.10 ± 0.05. There was no significant interaction between the event (first and second compensatory step TD) x group (AC and Stroke survivors) for X_COM/BOS_ [F_1, 21_ = 0.02, *p* = 0.89] and V_COM/BOS_ [F_1, 21_ = 0.75, *p* = 0.39] (Figure 7(a, b)). For X_COM/BOS,_ there was no significant main effect of event [F_1, 21_ = 0.07, *p* = 0.78] and main effect of group [F_1, 21_ = 0.22, *p* = 0.64]. However, for V_COM/BOS_ there was a significant main effect of event [F_1, 21_ = 4.63, *p* = 0.04] and a significant main effect of group [F_1, 21_ = 9.08, *p* = 0.007]. The second compensatory step length was significantly longer in stroke survivors than the AC group (*p* = 0.02, 95% CI = −0.13, −0.008) (Figure7(c)).

**Figure 8.**
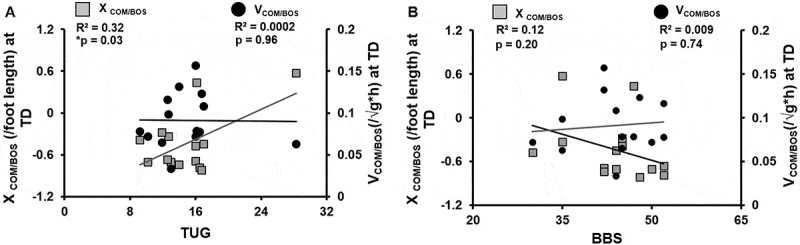
Scatter plot illustrating relationship of (a) Timed-up and go (TUG) test with XCOM/BOS and VCOM/BOS at touchdown (TD). (b) Berg balance scale (BBS) score with XCOM/BOS and VCOM/BOS at TD for stroke survivors. Significance is indicated by * *p* < 0.05 and ** *p* < 0.01

Within the stroke survivors, TUG score was significantly predicted by X_COM/BOS_ at TD (*R*^2^ = 0.32, *p *= 0.03). A significant positive correlation was found between TUG and X_COM/BOS_ at TD (*r* = 0.56, *p* = 0.03). However, no significant relationship was observed between TUG and V_COM/BOS_ at TD (*r *= −0.01, *p* = 0.96) (Figure8(a)). There was no significant correlation between BBS and X_COM/BOS_ at TD (*r *= −0.36, *p* = 0.20); BBS and V_COM/BOS_ at TD (*r *= 0.09, *p* = 0.74) (Figure8(b)). Further, there was no relationship between CMSA and COM state at TD, X_COM/BOS:_ (r_s_ = −0.19, *p* = 0.50) and V_COM/BOS:_ (r_s_ = 0.23, *p* = 0.41).

## Discussion

The results of this study demonstrated a reduced reactive control of the COM state (X_COM/BOS_ and V_COM/BOS_) in the stroke survivors compared with the age-similar older and younger controls for recovery from trip-related responses. Such reduced COM control along with impaired trunk control and reduced ability of the paretic limb to provide vertical limb support could affect the reactive trip-recovery responses and increase fall-risk in stroke survivors.

### First compensatory step

All participants in response to external perturbation demonstrated a FLOB. Thus, at LO they exhibited a X_COM/BOS_ beyond the anterior margin of the tripping/stance limb [significantly more positive X_COM/BOS_]. The subjects also had a COM that was traveling forward with a faster velocity than the BOS [more positive V_COM/BOS_]. Except for one participant among the stroke survivors, none experienced falls. From LO to TD, an improvement in X_COM/BOS_ was recorded for all groups, indicated by a negative X_COM/BOS_ at TD, which resulted from execution of a compensatory step that reestablished the BOS such that the COM lay posterior to it. Similarly, for all groups, at TD the V_COM/BOS_ was less positive indicating that the COM velocity was decelerating relative to the BOS at TD when compared with LO. However, for both the variables the stroke survivors still demonstrated a significantly more anterior and faster COM velocity than the YC.

Fall risks can be reduced by effectively modulating the compensatory step length (Wang et al. 2012), which is essential for reestablishing control of the COM state (X_COM/BOS_ and V_COM/BOS_) (Wang et al. 2012). In addition, controlling and reversing the trunk excursion following a trip is essential for preventing a fall (Honeycutt et al. 2016). In fact, in older adults it is observed that, greater trunk flexion is associated with lower postural stability during trips, contributing to increased fall risk (Crenshaw et al. 2012)

We noted that at first compensatory step TD, an improved relative X_COM/BOS_ (more negative values of X_COM/BOS_) was associated with greater step lengths, for all the three groups (YC, AC and stroke survivors). However, a significant relationship between trunk angle and X_COM/BOS_ only existed in the young group, where lower trunk flexion values were associated with more negative values of the X_COM/BOS_. The stroke survivors demonstrated the lowest correlation between X_COM/BOS_ and trunk angle. Further stroke survivors were more unstable demonstrating increased trunk flexion compared with the YC group at TD. Such findings indicate that may be the stroke survivors were attempting to compensate for impaired trunk control by appropriately modulating step length as they had the strongest correlation between X_COM/BOS_ and step length. Nonetheless the decreased control in X_COM/BOS_ and V_COM/BOS_ demonstrated at TD could have led to continued postural instability in stroke survivors resulting in the need to take additional compensatory steps, further predisposing them to a higher risk for falls. These findings are in line with our previous study that demonstrated that stroke survivors are unable reestablish stability with more than one step during backward loss of balance (Salot et al. 2016).

In the current study, even though majority of the stroke survivors stepped with their unaffected limb their first compensatory step was significantly delayed as compared with the YC and AC groups. It is known that among stroke survivors, the weight-bearing asymmetry favoring the non-paretic limb still persists even in the chronic phases of recovery (Tasseel-Ponche et al. 2015). Thus, due to the greater loading of the unaffected limb along with their difficulty in transferring the body mass to the paretic limb could have affected the unloading of the non-paretic limb causing delays in initiating the compensatory step (Lakhani et al. 2011; Inness et al. 2014). Such delayed step initiation might have contributed to greater instability (observed by a more anterior X_COM/BOS_ and V_COM/BOS_ at LO) among stroke survivors at LO compared with their healthy counterparts.

### Vertical limb support

In addition to a large compensatory step, adequate limb support assists in generating the required joint moment to counter the balance loss and prevent a vertical collapse during balance recovery (Pijnappels et al. 2004; Pai et al. 2006). Furthermore, the support limb also provides sufficient time and clearance for appropriate positioning for stepping (recovery limb) at TD to restrain the body from moving forward (trunk control) (Pijnappels et al. 2008) thus, maintaining an upright posture. Although the first compensatory step length in the stroke survivors was similar to the control groups (YC and AC), the inability to provide adequate vertical limb support could have been instrumental in the continued FLOB in this group. The event of peak hip descent [indicator of net vertical limb support torque and linearly correlated to vertical ground reaction force (Yang et al. 2009)] occurred, subsequent to the first compensatory step touchdown in this group [peak Z hip time = 1.68 (0.65) s and first TD time = 1.65 (065) s]. Considering the delayed step initiation and a more anterior COM state at LO (X_COM/BOS_ and V_COM/BOS_) demonstrated in the stroke survivors than the other groups. Stroke survivors might have required a larger first step length than the healthy control groups.

### Age-related differences and second compensatory step

With regards to AC group, their control of X_COM/BOS_ and V_COM/BOS_ post TD of the first compensatory step tended to be lower than the YC, but greater than the stroke survivors. Despite this, similar to the stroke survivors, majority of the age-similar controls also demonstrated multiple stepping. It is possible that they adopted multiple stepping strategy due to an age-related decline in balance function and fear of falling which are common with advancing age. Previous studies in healthy older adults have confirmed the age-related decline in musculoskeletal and sensorimotor function (McIlroy and Maki 1996; Maki and McIlroy 2005) and psychological factor such as fear of falling (Schulz et al. 2005) to be the underlying contributors for multiple stepping in response to external perturbation. Additionally, studies have also concluded that healthy older adults demonstrate a tendency to exhibit additional steps when not imperative (Mille et al. 2003; Carty et al. 2011).

Thus following the second compensatory step in the AC group, although their X_COM/BOS_ at TD did not differ as compared with first step TD, their V_COM/BOS_ was significantly lower than the first compensatory step TD. The AC group was able to control their V_COM/BOS_ to reduce instability in the forward direction. The compensatory step not only serves to restore or expand the BOS but at TD of the foot there is a counterclockwise torque exerted which can decelerate the forward momentum of the COM (i.e. reduce its forward velocity). The stroke survivors, who stepped with their paretic limb, however, unlike the AC group, were unable to control V_COM/BOS_ despite longer compensatory step length than the AC group. It is possible that due to stroke-induced impairment, even a long second compensatory step was not efficient in providing adequate limb support to generate an enough upward moment to reduce the COM velocity and reverse the already initiated hip descent (which peaked between the first and second compensatory step TD as mentioned above).

In summary, the biomechanical findings from this study suggest that stroke-induced reduced ability of the paretic limb to generate adequate vertical support (initially in its role as the stance limb and subsequently in its role as the stepping limb during the second compensatory step) and to control forward trunk rotation could be key factors predisposing stroke survivors to an elevated fall-risk.

## Clinical scales and reactive postural control

In the current study, stroke survivors demonstrated a mean score of 43.6 out of 56 for BBS and an average time-period of 15 s on TUG. Therefore, it implies that the stroke survivors in our study were at low risk of falls and had good independent mobility. However, these individuals failed to demonstrate good postural control and sufficient stability at the first step TD in response to a sudden trip like perturbation. Further the postural control measures on the first trip-like perturbation did not show a strong relationship with the routinely used clinical measure of BBS and the CMSA motor impairment scale. Similar to our findings a previous slip perturbation study has shown clinical tests to be poor predictors of fall risks in older adults (Boulgarides et al. 2003; Laessoe et al. 2007; Santos et al. 2011), with TUG being the only clinical scale moderately related to fall-risk prediction (Bhatt et al. 2011). Previous findings have also documented a potential limitation of many clinical measures of balance, mobility, or limb control in identifying fall-risk, as these measures evaluate volitional limb control and self-governed speed of movement (Boulgarides et al. 2003; Harris et al. 2005), which might be fundamentally different from the control and speed required for reactive stepping. Further many of these scales have ceiling effects and lack the sensitivity which instrumented measures can provide (Blum and Korner-Bitensky 2008; Lundin-Olsson 2010; Pardasaney et al. 2012).

### Trip versus slip recovery

Interestingly, during trip-like stance perturbations, we observed very low incidence of falls among stroke survivors. In contrast, our previous study examining responses to slip-like stance perturbations showed a higher falls incidence (i.e. >50% at similar perturbation intensity) (Patel and Bhatt 2016; Salot et al. 2016). It is likely that stroke survivors demonstrate greater balance control during forward compared with a backward balance loss from standing position. Such observations have been made in other studies in healthy adults as well during gait perturbations suggesting a possible role of perturbation direction in the ability to prevent a fall (Grabiner et al. 2008).

When compared with recovery from a slip-like perturbation (Patel and Bhatt 2016; Salot et al. 2016), a trip-like perturbation involving forward stepping (Dijkstra et al. 2015) simulates a motor behavior similar to walking. Normal walking requires forward rhythmic progression leading to a long-term adaptation in limb coordination, muscle memory and neural pathways for such activity (Pearson 2000). It is proposed that the neuronal network responsible for rhythmic motor patterns can be altered and strengthened by input from afferent sensory motor inputs (Pearson 2000). Further, any changes in afferent inputs could modify the motor behavior (Yanagihara et al. 1993), possibly through changes in neural pathways (Pearson 2000). The stroke survivors in the current study were community ambulators and therefore, repetition of forward stepping movement during walking possibly strengthened the motor memory and neural pathway underlying lower limb coordination for forward stepping. Consequently, the motor memory for walking possibly assisted in maintaining postural control during forward compensatory stepping response elicited by trip-like perturbation.

## Conclusion and clinical implications

Stroke survivors demonstrated poor limb support and required more than one compensatory step to re-gain balance when compared with healthy young and age-similar older adults. The stroke survivors showed impaired ability to control COM state stability (X_COM/BOS_ and V_COM/BOS_) and trunk angle compared with the YC group at touchdown of the first compensatory step. Furthermore, at the second step touchdown the stroke survivors were unable to regain stability compared with healthy age-similar older adults, as the stroke survivors demonstrated reduced control of V_COM/BOS_ despite longer step length than age-similar healthy older adults. These deficits observed in the reactive recovery response in stroke survivors might contribute towards a higher fall-risk in this population.

Further research comparing the differential fall risk and recovery mechanisms to both forward and backward perturbations would provide deeper insight into likely causes of falls. Clinicians should consider incorporating compensatory step testing and training to externally-induced unanticipated perturbations for fall-risk assessment and intervention, among older stroke survivors, especially after they achieve independent standing and ambulation, since both age and stroke-induced impairments in reactive balance could predisposes this population to a significantly heightened fall-risk.
